# Would transcranial direct current stimulation (tDCS) enhance the effects of working memory training in older adults with mild neurocognitive disorder due to Alzheimer’s disease: study protocol for a randomized controlled trial

**DOI:** 10.1186/s13063-015-0999-0

**Published:** 2015-10-24

**Authors:** Calvin P. W. Cheng, Sandra S. M. Chan, Arthur D. P. Mak, Wai Chi Chan, Sheung Tak Cheng, Lin Shi, Defeng Wang, Linda Chiu-Wa Lam

**Affiliations:** Department of Psychiatry, Tai Po Hospital, 9 Chuen On Road, Tai Po, Hong Kong; Department of Psychiatry, The Chinese University of Hong Kong, Rm G25, G/F, Multi-center, Tai Po Hospital, 9 Chuen On Road, Tai Po, Hong Kong; Department of Psychiatry, The University of Hong Kong, 3/F, HKJC for Interdisciplinary Research, 5 Sassoon Road, Pokfulam, Hong Kong; Department of Health and Physical Education, Hong Kong Institute of Education, Rm D4-2/F-03, Block D4, 10 Lo Ping Road, Tai Po, NT Hong Kong; Department of Medicine and Therapeutics, The Chinese University of Hong Kong, 9/F, Lui Che Woo Sciences Building, Prince of Wales Hospital, Shatin, Hong Kong; Department of Imaging and Interventional Radiology, The Chinese University of Hong Kong, Prince of Wales Hospital, Shatin, Hong Kong

**Keywords:** Alzheimer’s disease, Brain stimulation, Cognitive function, Neurocognitive disorder, Randomized clinical trial, Transcranial direct current stimulation, Working memory

## Abstract

**Background:**

There has been longstanding interesting in cognitive training for older adults with cognitive impairment. In this study, we will investigate the effects of working memory training, and explore augmentation strategies that could possibly consolidate the effects in older adults with mild neurocognitive disorder. Transcranial direct current stimulation (tDCS) has been demonstrated to affect the neuronal excitability and reported to enhance memory performance. As tDCS may also modulate cognitive function through changes in neuroplastic response, it would be adopted as an augmentation strategy for working memory training in the present study.

**Methods/Design:**

This is a 4-week intervention double-blind randomized controlled trial (RCT) of tDCS. Chinese older adults (aged 60 to 90 years) with mild neurocognitive disorder due to Alzheimer’s disease (DSM-5 criteria) would be randomized into a 4-week intervention of either tDCS-working memory (DCS-WM), tDCS-control cognitive training (DCS-CC), and sham tDCS-working memory (WM-CD) groups. The primary outcome would be working memory test – the n-back task performance and the Chinese version of the Alzheimer’s Disease Assessment Scale – Cognitive Subscale (ADAS-Cog). Secondary outcomes would be test performance of specific cognitive domains and mood. Intention-to-treat analysis would be carried out. Changes of efficacy indicators with time and intervention would be tested with mixed effect models.

**Discussion:**

This study adopts the theory of neuroplasticity to evaluate the potential cognitive benefits of non-invasive electrical brain stimulation, working memory training and dual stimulation in older adults at risk of cognitive decline. It would also examine the tolerability, program adherence and adverse effects of this novel intervention. Information would be helpful for further research of dementia prevention studies.

**Trial registration:**

ChiCTR-TRC-14005036 Date of registration: 31 July 2014.

## Background

The World Alzheimer’s Report and World Health Organization (WHO) have highlighted dementia care as a public health priority [1, 2]. Despite increased understanding of putative pathological mechanisms, treatment options for dementia remains limited. Recent research has suggested that factors that influence the integrity and function of neuronal networks may help to resist the emergence of disabling clinical symptoms. Interventions that enhance neuroplastic response through stimulation may impact on cognitive impairments and possibly attenuate the trajectories of decline.

Cognitive impairment is the core syndrome of dementia. It also determines the loss of independent functioning. There has been long-standing interest as to whether cognitive training, programs with structured training of specific cognitive domains would help to improve cognition and function in people suffering from dementia [3–5]. In a recent study and a systematic review, it appeared that there was limited improvement in cognitive functions or behavior measures associated with cognitive training in people with dementia [6, 7]. However, other recent studies suggested a possibility of transferring cognitive benefits with working memory training paradigms. A working memory (WM) training task, the Adaptive n-back task, has recently been reported to offer improvements in cognitive domains other than working memory, including episodic memory and other measures of fluid intelligence [8, 9]. Improvements in performance were also found in older adults and were more marked in people with lower baseline performance [10]. This suggested that the n-back training has a potential to enhance cognitive reserve, whereas individual differences may be modulated by one’s capacity for neuroplastic response. However, replication studies published in 2013 were less promising. Transferrable cognitive benefits of n-back training beyond working memory tasks have not been demonstrated [11, 12]. Apart from differences in methodological designs, it is possible that cognitive training is able to effect overall improvement, but the transformation of underlying neuronal networks has been unstable. Before concluding that WM training is not beneficial for overall cognitive improvement, it is important to explore strategies that may augment and consolidate cognitive benefits.

Recent research suggests that non-invasive brain stimulation might enhance cognitive function [13]. Transcranial direct current stimulation (tDCS) is one promising option for exploration. The tDCS device generates a small electric current (usually 1 mA to 2 mA) to specific areas of the brain, and alters excitability of brain cells through polarity-dependent stimulation. Anodal stimulation of the brain areas generally enhances excitability, whereas cathodal stimulation reduces it. The manipulation of electrode positions and polarity would lead to different regional effects in brain areas. Anodal stimulation of the dorsal lateral prefrontal cortex (DLPFC) and temporal areas have been reported to enhance memory performance in healthy young and older-aged volunteers. In people suffering from Alzheimer’s disease (AD), recognition memory also appeared to improve after tDCS and effects persisted for a few weeks after intervention. A functional magnetic resonance neuroimaging study (fMRI study) reported that default mode network (DMN) activities in older adults showed a tendency for reversal to the pattern of the young adult volunteers after tDCS. This suggested possible physiological effects of enhanced neuroplastic response [14–19]. As for the safety and adverse event profiles, no major or serious adverse effects of tDCS had been reported in the past decade of research. The risk of inducing seizure was minimal.

Available evidence suggests that cognitive training is able to improve cognitive performance, at least in the domain being trained. Specifically designed working memory training may have an additional role of transferrable cognitive benefits. This suggested that our brain is responsive to mental stimulation with enhanced function. Electrical stimulation to the brain appeared to alter neuronal activities with corresponding changes in cognitive and other brain functions. It is possible that changes in neuroplasticity brought about by electrical stimulation would modulate the neural response to mental stimulation through cognitive training. The present study aims to examine the combined longer-term effects of tDCS working memory training, compared to single-modality intervention of either tDCS or WM, on cognition function.

## Methods/Design

A 4-week intervention (12-week observation) double-blind randomized controlled trial (RCT) of tDCS. Participants will be randomized into three groups:

1. The working memory (Adaptive n-back) training-sham tDCS group (WM-CD).
  Participants would receive 20 minutes of sham tDCS, followed by 5 minutes of rest and 20 minutes of Adaptive n-back training.
2. The tDCS-control cognitive training group (DCS-CC).
  Participants would receive 20 minutes of tDCS, followed by 5 minutes of rest and 20 minutes of controlled cognitive training with continuous performance tasks.
3. The tDCS-working memory training (DCS-WM) group.
  Participants would receive 20 minutes of tDCS, followed by 5 minutes of rest and 20 minutes of Adaptive n-back training.

The intervention would be of 4 weeks duration, with 3 training sessions (45 minutes) per week. All groups would receive the same training schedule.

### Participants and recruitment

Participants would be recruited through existing research cohorts and local advertisements at older persons’ social centers in Hong Kong. Informed consent would be obtained from each eligible participant. Interested participants would first undergo a cognitive and clinical assessment for mild neurocognitive disorder due to AD (MND-AD) before recruitment for intervention. The cognitive assessment was derived from previous studies of the research team [20]. Potential participants would be assessed by psychiatrists of the research team for eligibility.

Inclusion criteria are as follows:

1. Subjects from 60 to 90 years old;
2. *Diagnostic and Statistical Manual of Mental Disorders, 5th edition* (DSM-5) diagnosis of MND-AD [18];

Exclusion criteria are set as:

1. Previous diagnosis of other major neurocognitive disorders;
2. Past history of bipolar affective disorder or psychosis;
3. Physical frailty affecting attendance at training sessions;
4. Already attending regular cognitive training;
5. Taking a psychotropic or other medication known to affect cognition (e.g. benzodiazepines, anti-dementia medication, etc.);
6. History of major neurological deficit including history of stroke, transient ischemic attack or traumatic brain injury;
7. Significant communicative impairments

### Aims and hypotheses to be tested

The present proposal aims to address the above question with a paradigm having high potential for clinical practice. The proposed neuroenhancement intervention combined the dual approach of mental and electrical stimulation. We hypothesized that

1. Adaptive n-back task training (with sham tDCS) would be associated with improved test performance in both working memory and other cognitive domains.
2. Stimulation to the right temporal cortex by tDCS (controlled cognitive training) would be associated with enhancement of test performance in memory and other cognitive domains. (Please refer to Intervention paradigms for details).
3. The effects of improvement in global cognitive domains would be comparable in single- modality brain stimulation and Adaptive n-back training or tDCS.
4. Combined tDCS and Adaptive n-back task training would be associated with more significant improvement in cognitive performance over single-modality brain stimulation by either Adaptive n-back task or tDCS.

### Randomization procedure

This will be a double-blind RCT of tDCS. Participants will be individually randomized into WM-CD, DCS-CC, or DCS-WM groups. Randomization with blocks of six would be adopted to ensure a balanced allocation of groups. The participants would be blind to the tDCS status. The assessors for clinical outcomes would be blinded to the randomization status. The staff who perform the intervention would not know the assessment results.

### Definition of outcome measures (Appendix 1)

Primary outcomes

1. Working memory test – the n-back task performance at baseline, fourth, eighth and twelfth weeks would be recorded as a direct measure of improvement in task performance.
2. Global Cognitive function would be measured by the Chinese version of the Alzheimer’s Disease Assessment Scale – Cognitive Subscale (ADAS-Cog). This is a standard global cognitive assessment for clinical intervention of AD. The scale score ranges from 0 to 70, with increasing scores indicating higher severity of global cognitive impairment [21].

Secondary outcomes

1. Memory and language tests – logical memory, 10 minutes list learning delay recall, category verbal fluency and trail-making tests would also be evaluated to examine the effects of both tDCS and working memory training on other cognitive abilities [22].
2. The Chinese Neuropsychiatric Inventory (NPI) [23] would be used to assess changes in neuropsychiatric symptoms across different twelve domains. In the current study, NPI would evaluate potential mood and behavioral change, especially mood and euphoria that may theoretically be affected by tDCS administration.
3. A checklist of potential adverse effects associated with computer-based cognitive training and t-DCS administration would be generated from available literature reports. The checklist would be used to monitor tolerability and adverse events throughout intervention.

### Intervention paradigms

1. Working memory (Adaptive n-back) and Control cognitive training (Appendix 2)

n-back task is a continuous performance test proposed by Kirschner for measurement of working memory capacity. During the n-back task, subjects are usually presented with a sequence of stimuli, and required to indicate which stimulus matches the one from n steps earlier in the sequence. Adaptive n-back task refers to automatic adjustment of the level of difficulty of the task according to the accuracy of the performance. Participants with high degree of accuracy would be advanced to another level of difficulty, whereas a suboptimal performance would be adjusted to an easier practice level [24, 25]. The Adaptive n-back visual training paradigm (WM) is programed using the E-Prime. The adaptive training offers a feedback mechanism and has a higher demand for attention throughout the training session. As the participants are older adults with little previous computer experience and may be more limited in working memory capacity, visual stimuli would be offered for training at the initial five levels. The dual task with auditory-visual stimuli would be offered after the participant has passed the 6-back training. It is considered that this approach would ensure higher motivation for long-term practice.

The Control cognitive training (CC) would be a continuous performance test paradigm. This is considered as an active control as the paradigm demands sustained attention similar to the n-back task. However, it does not offer training of the working memory component embedded in the n-back task.

2. Transcranial direct current stimulation (tDCS)

In each session, tDCS (DCS) would be administered with a current strength of 2 mA for 20 minutes (NeuroConn, DC-stimulator Plus, http://www.neuroconn.de/kontakt_en/). The decisions regarding the location of stimulus and electrodes are made with reference to a recent fMRI DMN study of Chinese older adults with mild cognitive impairment (MCI) conducted by the project team. In our study, subjects with MCI were found to have reduced activity of the left temporal lobe, but with increased activity of the right frontal lobe (Shi et al., submitted). Anodal stimulation would be applied to the left temporal cortex, positioned over T3 and T4 according to the 10–20 electroencephalogram (EEG) international system. The reference electrode would be place over the right deltoid muscle. The selection of non-cephalic reference electrode is to avoid interference of compensatory brain networks of the subjects with MCI. The electrodes used for tDCS were 35 cm^2^ in area [26]. The sham tDCS (CD) schedule would include 30 seconds of 2-mA electrical stimulation to the temporal cortex; the electric current would then stop while the device would be retained until the end of the 20-minute period. Afterwards, the subject would rest for 5 minutes before commencement of 20 minutes of Adaptive n-back task.

### Statistical analyses

Multilevel generalized linear modeling would be employed to account for the correlations from within subjects and different time points of measurement. Baseline cognitive characteristics between tDCS-working memory training (DCS-WM), tDCS-control cognitive training group (DCS-CC) and working memory training–-ham tDCS (WM-CD) groups would be evaluated. Changes of cognitive function and behavioral symptoms from baseline to each follow-up point and intervention differences would be tested with occasions (time points) at level 1 and subjects at level 2. Global cognitive function between DCS-WM, DCS-CC would be made with WM-CD (reference group). Covariates including baseline differences would be entered in the regression model. Secondary analyses of specific cognitive function would be performed to compute for group differences in outcome. Incidence of adverse events and characteristics of program adherence would be recorded. Statistical significance will be set at *p* < 0.05. Bonferroni corrections would be adopted for adjustment of multiple comparisons. Computations would be performed using SPSS (SPSS Inc., Chicago, IL, USA) for windows version 20.0 and Stata (StataCorp, College Station, TX, USA).

### Sample size estimation

The sample size is estimated with using GPower 3.1. Measurements would be evaluated comparing the cognitive test performance across time and intervention groups. The potential effect size of cognitive enhancement of the cognitive training and tDCS is estimated from the findings of a cognitive activity intervention conducted with older adults with MCI in Hong Kong (2012). After 12 weeks, the 3 sessions of weekly cognitive activity intervention was associated with improvement in ADAS-Cog (11.3 ± 3.2 versus 8.8 ± 3.5), and delay recall tests (3.5 ± 2.3 versus 5.8 ± 2.1) (Cheng et al., unpublished data). Sixteen subjects in each group will be required to achieve a power of 0.8 in detecting improvements with intervention. Assuming a medium effect size of 0.5 of cognitive enhancement at the 12th week in dual stimulation over single-modality intervention, 51 subjects are required in each group. Taking into account the dropout rate of 25 %, 64 participants per arm should be recruited.

### Ethical consideration

A psychogeriatrician who is familiar with mental capacity assessment would assess mental capacity for participation in this research. Participants would be recruited if they are considered mentally fit to sign consent. First degree relatives of participants will also be informed before intervention. Ethics approval from The Joint Chinese University of Hong Kong – New Territories East Cluster Clinical Research Ethics Committee has been obtained, and the protocol registered with the Center for Clinical Research and Biostatistics, Clinical Trials Registry of the Chinese University of Hong Kong and linked to the Chinese Clinical Trials Registry (ChiCTR), World Health Organization – International Clinical Trials Registry Platform (WHO-ICTRP) China Primary Registry (ChiCTR-TRC-14005036). The study will comply with the Declaration of Helsinki and the Good Clinical Practice guidelines of the International Conference on Harmonization of technical requirements for registration of pharmaceuticals for human use (ICH-GCP). The results will be reported according to Consolidated Standards of Reporting Trials (CONSORT) Guidelines for non-pharmacological interventions.

## Discussion

The present study aims to examine the cognitive effects of working memory training in older adults with MND-AD, and to evaluate whether the effects would be transferrable to other cognitive domains. The findings, if positive, would provide a basis for enhancement of cognitive reserve through brain stimulation. Second, we would also evaluate the cognitive effects of tDCS. The information would help to address the impact of electrical stimulation on brain function, which would bring insights into therapeutic potential of other psychiatric disorders associated with cognitive dysfunction. More importantly, we hope to examine if adjuvant electrical stimulation would consolidate the brain response through manipulation of neuroplasticity. This would provide directions for research in the neurophysiological basis of synergistic brain stimulation of different modalities.

From a clinical perspective, the current proposal would serve as a Phase 2 non- pharmacological intervention. It would examine the potential efficacy of cognitive training, electrical brain stimulation, and dual mental and electrical brain stimulation. The proposed study would also examine the tolerability, program adherence and adverse effects of this intervention in older adults. As stipulated by a recent report by the Alzheimer’s Australia, an intervention that delays the onset by 2 years will reduce the prevalence of dementia by 13 % in 3 decades [27]. If the findings suggest potential positive benefits, and with low risks, the proposed intervention would provide a solid basis for further evaluation by further Phase 3 clinical trials with implementation in clinical settings for dementia prevention.

## Trial status

Recruitment started in March 2015. Recruitment is ongoing.

## Figures and Tables

**Fig. 1:**
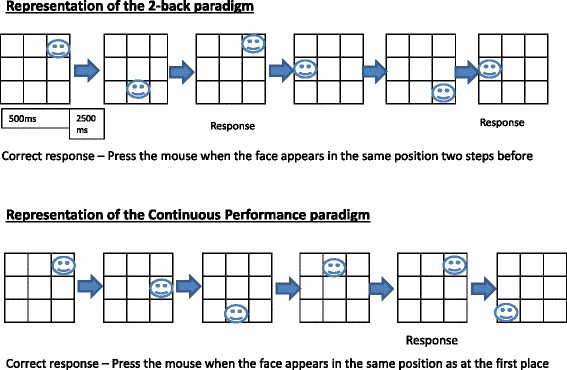
Cognitive training paradigms (3 second trials – 20 trials per block for 20 blocks)

## Appendix

### Appendix 1

**Table 1:** Assessment schedule

	Baseline assessment	First follow-up (4th week)	Second follow-up (8th week)	Third follow-up (12th week)
ADAS-Cog	V	V	V	V
Cantonese MMSE	V	V	V	V
n-back test performance	V	V	V	V
Delay recall	V	V	V	V
Logical memory	V	V	V	V
Digit and Visual Span tests	V	V	V	V
Category Verbal Fluency Test	V	V	V	V
Trail-making test	V	V	V	V
Neuropsychiatric Inventory	V	V	V	V
Adverse event checklist	V	V	V	V

*ADAS-Cog* Alzheimer’s Disease Assessment Scale – Cognitive Subscale, *MMSE* Mini-mental State Examination

## Abbreviations

AD: Alzheimer’s disease
ADAS-Cog: Alzheimer’s Disease Assessment Scale – Cognitive Subscale
CC: Control cognitive training
ChiCTR: Chinese Clinical Trials Registry
CONSORT: Consolidated Standards of Reporting Trials
DCS-CC: tDCS-control cognitive training
DCS-WM: tDCS-working memory training
DLPFC: dorsal lateral prefrontal cortex
DMN: default mode network
DSM-5: *Diagnostic and Statistical Manual of Mental Disorders, 5th edition*
EEG: electroencephalogram
fMRI: functional magnetic resonance imaging
ICH-GCP: Good Clinical Practice guidelines of the International Conference on Harmonization
MCI: mild cognitive impairment
MoCA: Montreal Cognitive Assessment
NPI: Neuropsychiatric Inventory
RCT: randomized controlled trial
tDCS: transcranial direct current stimulation
WHO: World Health Organization
WM: working memory
WM-CD: Sham tDCS-working memory
